# Trend of C-Reactive protein after colorectal surgery may increase predictive value of anastomotic leak: a prospective analysis

**DOI:** 10.1186/s12893-026-03511-0

**Published:** 2026-03-05

**Authors:** Salvatore Tramontano, Umberto Bracale, Biancamaria Iacone, Luigi Schiavo, Antonio Gargiulo, Anna Mirea Tedesco, Luigi Ricciardelli, Francesco Corcione

**Affiliations:** 1 General and Emergency Surgical Unit, Fucito Hospital, San Giovanni di Dio e Ruggi d’Aragona University Hospital, Salerno, Italy; 2 Department of Medicine, Surgery and Dentistry “Scuola Medica Salernitana”, University of Salerno, Baronissi, Italy; 3 Department of Public Health, University of Naples Federico II, Naples, Italy; 4 Emergency Surgical Unit, AORN Ospedale dei Colli, Naples, Italy; 5 Oncological and mini-invasive Surgery, Mediterranean Clinic, Naples, Italy

**Keywords:** C-Reactive protein, Colorectal surgery, Anastomotic leak

## Abstract

**Introduction:**

Early diagnosis of anastomotic leak (AL) after colorectal surgery is essential to reduce mortality and overall survival. C-reactive protein (CRP) is among the factors most significantly associated with infection and AL. Analysis of trend in CRP levels could be crucial in increasing its predictive value.

**Aims:**

We performed a prospective evaluation on a cohort of patients undergoing colorectal resection for cancer, comparing CRP values on the first postoperative days to assess delta CRP in relation to identification of AL.

**Methods:**

Our prospective evaluation considered cases of elective colorectal surgery for cancer, enrolled by two high-volume centers from January 2021 to June 2024. We considered 2 groups: leakage (L group) and non-leakage (NL group). Evaluation of CRP levels on POD 1 and 3 was recorded, analyzing linear difference (delta CRP). Patients with suspicion of AL underwent postoperative imaging.

**Results:**

Final analysis was conducted on 320 patients. Among these, 13 (4.1%) experienced an AL. CRP levels on POD1 and POD3, and delta CRP variables demonstrated AUC values of 0.692, 0.902, and 0.969, respectively, indicating that delta CRP is the most predictive measure compared to individual CRP values. Delta CRP reported a specificity of 0.964 and a sensitivity of 0.923.

**Conclusions:**

Our data confirmed that high NPV of postoperative CRP trend was able, already on POD3, to rule out anastomotic complications. CRP trend between POD1 and POD3 seems to increase the sensitivity and specificity of this biochemical marker, which should be confirmed by larger cohorts, considering this a pilot study.

**Supplementary Information:**

The online version contains supplementary material available at 10.1186/s12893-026-03511-0.

## Introduction

Anastomotic leak (AL) is considered the most severe complication of colorectal surgery. It is related with worse prognosis of oncological patients and is associated with mortality over 30% [1]. AL is generally defined as a failure of anastomotic closure, with communication or extravasation of intraluminal contents [2]. In some cases, the presence of abscess near the site of anastomosis is included in diagnosis of AL [3]. The incidence of AL varies from 1% to 30% [1, 4]. This variation in the incidence rate is attributed to the variety of definitions of anastomotic leakage found in the medical literature [4]. Early diagnosis is essential to reduce mortality, length of hospital stay, postoperative complications, costs, and overall survival. Different scores were developed to help identify patients at high risk for this complication [2, 4]. Diagnostic tools for AL are computed tomography (CT), abdominal drain secretion analysis and, in some cases, endoscopic examination. Biomarkers are often more sensitive for early detection of AL and are useful to confirm imaging diagnosis [2, 5]. Clinical status may vary widely, however, and it is often not related with early detection of AL [6]. Surgical intervention is frequently necessary. Currently, available evidence indicates a prognostic value for several proteins associated with the inflammatory response in the early detection of patients with AL following colorectal resection [7]. The cutoff and timing for assessing these parameters have not yet been standardized in the literature, despite their solid statistical validity. In particular, C-reactive protein (CRP) is among the factors most significantly associated with infection and AL in colorectal surgery, with sensitivity over 80% in various studies [8, 9]. CRP is one of the most widely studied biomarker, and was first described in 1930 by Willian S. Tillet and Thomas Francis [10]. It is an acute-phase protein with an early pattern of secretion, after inflammatory stimulus, reaching peak in the plasma at 48 h. Its trend well reflects inflammatory course [11]. In addition, its short half-life (19 h), makes CRP a reliable marker following surgical procedure, mostly with anastomosis. Moreover, concomitant pathologies and associated complications or interindividual variability may affect sensitivity of this marker for detection AL [11, 12]. In literature, variability of cut-off and association with complications confirms the need for standardization and statistical efforts [11–13]. Although the prognostic value of CRP is not currently in question, there are some limitations related to variability between patients, who often have comorbid conditions or a subclinical course of the fistula [11, 14].

A factor that is often overlooked in the literature is the trend in CRP levels, meaning the difference between immediate postoperative values and those during the hospital stay. This variation could be crucial in increasing the parameter’s predictive value, as it helps exclude initial biases related to, for example, pre-existing inflammatory states. The evolving trend of CRP could also help identify cases of subclinical fistulas that do not reach validated cutoffs and therefore would not be included in the high-risk fistula group.

In literature, the CRP trend has mainly been evaluated in patients undergoing surgery for chronic inflammatory bowel diseases, who often have higher baseline CRP values than the general population due to chronic inflammatory processes associated with the underlying condition [15]. In oncology, however, the purpose of monitoring the CRP trend is to detect an anastomotic complication early, without relying on absolute parameters but rather observing a deterioration in the clinical course, marked by pathological inflammatory response, which often underlies AL, the most common septic complication in colorectal surgery [16].

To the scope, we have started a prospective evaluation on a multicenter cohort of patients undergoing colonic or rectal resection for cancer, with the goal of comparing CRP values on the first and third postoperative days to assess the difference (delta CRP) in relation to the clinical or radiological identification of an anastomotic fistula. Aim of the analysis was identification of a possible higher statistical power of trend pf CRP than single evaluation on postoperative days. This should set a more sensitive postoperative analysis, with more biomarkers that may predict the most important complication of colorectal surgery, affecting on morbidity and mortality.

### Patients and methods

Our prospective evaluation included patients of both genders who underwent elective colorectal surgery for cancer, with primary anastomosis. Multicentric cohort was composed from January 2021 to June 2024, and included two surgical teams with high-volume colorectal cancer. A common database was shared at the beginning. Data of consecutive patients with histologically proven colorectal cancer undergoing elective surgery in the surgical units involved in the study were collected in an electronic database. Demographic and clinical data, including sex, age, body mass index (BMI), American Society of Anesthesiologists (ASA) score, Charlson comorbidity index (CCI), hospital stay, localization of the disease, as well as the stage of the disease according to the globally recognized TNM staging system, were registered. Preoperative CRP was considered as inclusion criteria. Furthermore, details regarding the surgical procedure, postoperative course, morbidity, and 30-day mortality were collected. Patients undergoing surgery with both an open or laparoscopic technique by experienced surgeons were included. Database also included nutritional, biochemical and inflammatory tests.

Patients who did not present at least serum CRP levels at first and third postoperative day (POD) were excluded. Other exclusion criteria were age lower than 18 years, surgery on an emergency setting, patients with previous ongoing infection (abdominal abscess and active enterocutaneous fistula) or current steroid therapy, and surgeries without an anastomosis. Patients that developed other infectious complications after surgery were also excluded. All the patients signed an informed consent for each procedure performed. The study was carried out in accordance with the principles of the Declaration of Helsinki.

The patients were divided into 2 groups, according to detection of AL during hospital stay: leakage (L group) and non-leakage (NL group). Standard diagnostic criteria of AL were detection of air of abscess near the site of anastomosis on ultrasound or CT; purulent or enteric secretion through the drain or skin incision; clinical signs of peritonitis. Antibiotics were restarted in patients with leakage. Patients developing other infectious complications, such as wound and urinary tract infection or pneumonia were included in the study and recorded. All these patients were excluded from statistical analysis, as possible bias. All anastomoses were made up with stapling devices; the choice of the technique as well as the type and dimension of the stapling device was made by the surgeon based on the localization of the disease and the anatomical conditions of the patients: latero-lateral anastomosis for right and splenic flexure cancer, termino-terminal for left and rectal cancer.

The groups were compared also for sex, age, BMI, ASA score, CCI, hospital stay, localization of the disease, for possible bias. Serum CRP levels were evaluated before surgery and every day from POD 1 to POD 5 for all patients through 7 immunoassays using the turbidimetric method with an Architect Plus C4000 analyzer (Abbot, Lake Bluff, IL, USA). CRP levels > 5 mg/L were considered altered. Specific evaluation on POD 1 and 3 was recorded, analyzing difference of absolute value at two measurements (defining as delta CRP). Daily clinical monitoring included abdominal pain, fever, return of bowel habits, and evaluation of abnormal secretion of drainage, if present. Patients with altered parameters or clinical suspicion underwent laboratory and imaging examinations (ultrasound or CT). All patients received antibiotics in prophylaxis and on first POD 1, and mechanical preparation of the colon was performed only for left and rectal cancer.

The linear difference between CRP level on POD 1 and 3 was calculated. Patients with suspicion of AL underwent postoperative imaging and received treatment in case of symptoms or complications. Clavien-Dindo classification was used to evaluate severity of AL. All reoperations performed were recorded.

### Statistics

Statistical analyses are performed using R software (version 4.3.1, released on June 16, 2023). Continuous variables are summarized as mean and standard deviation or as median and interquartile range, depending on the data distribution. The assumption of normality is assessed through graphical inspection and the Kolmogorov-Smirnov test. Categorical variables are reported as absolute frequencies and percentages.

Group comparisons for continuous variables are conducted using the Student t-test or the Wilcoxon rank-sum test, depending on the data distribution. For categorical variables, comparisons are performed using the chi-square test.

Predictive capacity of variables related to the CRP is evaluated through the construction of ROC curves. The optimal cut-off is determined by maximizing the Youden index. Confidence intervals (CIs) for sensitivity, specificity, positive predictive value (PPV), negative predictive value (NPV) and accuracy, are calculated using the epi.tests function from the epiR package. All reported CIs are at the 95% level and statistical significance is set at with a first-type error (α) of 5%.

## Results

A total of 406 patients undergoing surgery for colorectal cancer between January 2021 and June 2024 at two institutions were identified. Sixty-four patients with ongoing infection (including abdominal abscess and active enterocutaneous fistula), current steroid therapy prior to surgery, under- going emergency surgery or bowel resection with end- ileostomy were excluded. A subgroup of 22 patients with complications not related to AL were also excluded. After excluding ineligible patients, the analysis was conducted on a total of 320 patients. All patients performed standard preoperative preparation with oral antibiotics and endovenous infusion at induction. Intestinal preparation with Macrogol 4000 was also completed in all enrolled cases the day before surgery. Among these, 13 (4.1%) experienced an AL. Table 1 summarizes the baseline clinical characteristics and CRP values at different time points.

**Table 1:** Baseline antropometric and clinical characteristics and CRP values at different time points

	Overall	No Leak	Leak	*p*
BMI _kg/m2_ (median [IQR])	24.20 [22.39, 26.45]	24.22 [22.33, 26.48]	23.30 [22.90, 25.60]	0.585
Age _years_ (mean (SD))	70.83 (10.36)	71.07 (10.34)	65.38 (9.48)	0.053
Sex = M (%)	201 (62.8)	191 (62.6)	10 (76.9)	0.451
CRP pre-surgery mg/dL (median [IQR])	0.75 [0.20, 3.34]	0.76 [0.20, 3.39]	0.70 [0.20, 1.00]	0.735
Hospital stay (median [IQR])	6.00 [5.00, 7.18]	6.00 [5.00, 7.00]	11.00 [10.00, 15.00]	< 0.001
Charlson Comorbidity Index - CCI (median [IQR])	23.9 (11–29)	22.0 (12–28)	24.7 (12–29)	0.431
Cancer location (colon/rectum - n; %)	215/105; 67.2%/32.8%	176/115; 60.5%; 39.5%	18/11; 62.1%/ 37.9%	0.761
Right colon (n)	89	85	4	0.750
Transverse colon (n)	16	14	2	0.872
Left colon (n)	110	98	12	0,658
Laparoscopic/Open (n; %)	272/48 ; 85.0%/15.0%	249/42 ; 85.6%/14.4%	23/6 79.3%;20.7%	0.310
Conversion rate (n; %)	10; 3.6%	7; 2.8%	1; 4.3%	0,340
**TNM stage**				
T1	22; 6.8%	21; 7.2%	1; 3.4%	0.546
T2	104; 32.5%	100; 34.4%	4; 13.7%	0.789
T3	112; 35.0%	94; 32.3%	18; 62.1%	0.765
T4	82; 25.7%	76; 26.1%	6; 20.8%	0.321
N-	178; 55.6%	166; 55.3%	17; 58.6%	0.678
N+	142; 64.4%	130; 44.7%	12; 41.4%	0.233
M-	298; 93.1%	271; 93.1%	24; 82.7	0.435
M+	22; 6.9%	20; 6.9%	5; 17.3%	0.114
ASA score 3–4 (%)	42 (14.0)	31 (10.8)	11 (84.6)	< 0.001
POD 1 (median [IQR])	4.00 [2.00, 7.00]	4.00 [2.00, 7.00]	2.00 [1.00, 3.00]	0.018
POD 3 (median [IQR])	11.00 [6.93, 14.88]	10.90 [6.59, 14.00]	21.40 [16.85, 26.70]	< 0.001
Delta PCR (median [IQR])	6.00 [3.70, 8.12]	6.00 [3.50, 8.00]	19.40 [15.30, 23.70]	< 0.001

The variables BMI, preoperative CRP, POD1 CRP, and POD3 CRP did not follow a normal distribution and were thus described using medians and interquartile ranges (IQR). The median age was 70.8 years and 62.8% of patients were male. The median BMI was 24.2 kg/m^2^. Before surgery, 97 patients (30.3% of enrolled patients) received neoadjuvant radiotherapy and chemotherapy. All operations were completed without perioperative deaths, and the mean operation time was 231.9 ± 82 min. One hundred five patients (32.8%) had rectal cancer, and 215 patients (67.2%) had colon cancer. Prophylactic ileostomy was performed in 118 patients (36.9%). The use of abdominal drainage, neoadiuvant therapy and preoperative CRP did not affect the onset of leakage (*P* = 0.694; *P =* 0.590; *P =* 0.711). Anastomoses were all performed with mechanical anastomosis, according to standard technique. Latero-lateral anastomosis was performed in 204 cases (63.8%), and termino-lateral in 116 cases (36.2%).

The overall morbidity rate complications, except for AL, was 9.1% of the entire cohort, including 29 patients, 13 with surgical site infection, 9 with hemorrhagic complications, 2 with evisceration or stoma prolapse, and 5 with pneumonia. All were excluded from study.

AL was diagnosed between PODs 3 and 11 (median, 5.8 days). The median hospital stay was significantly longer in the L group than in the NL group (15 vs. 7 days; *P* < 0.001). CT was used to diagnose AL in 11 cases (84.6% of patients). All patients with AL underwent surgical treatment, including 2 (15.4%) Hartmann’s colectomies, 10 (76.9%) abdominal lavage with or without ileostomy, and one (7.6%) colorectal resection and reanastomosis. Clavien-Dindo classification was IIIb for 7 cases (77.8%) and IVa for 2 cases (22.2%). No difference was observed for IIIb and IVa cases in terms of delta CRP. No statistically significant differences were observed between L and NL group for the variables BMI, sex, preoperative CRP, age, CCI, hospital stay, and operative time. Oncological therapy also did not differ among groups (as evidenced in Table 1).

However, age demonstrated a p-value approaching 0.05, with a mean difference of approximately 6 years (L group being younger). While this finding might be incidental, it could suggest a potential factor to consider in future studies with larger sample sizes aimed at developing more accurate predictive models for leak events.

**Fig. 1:**
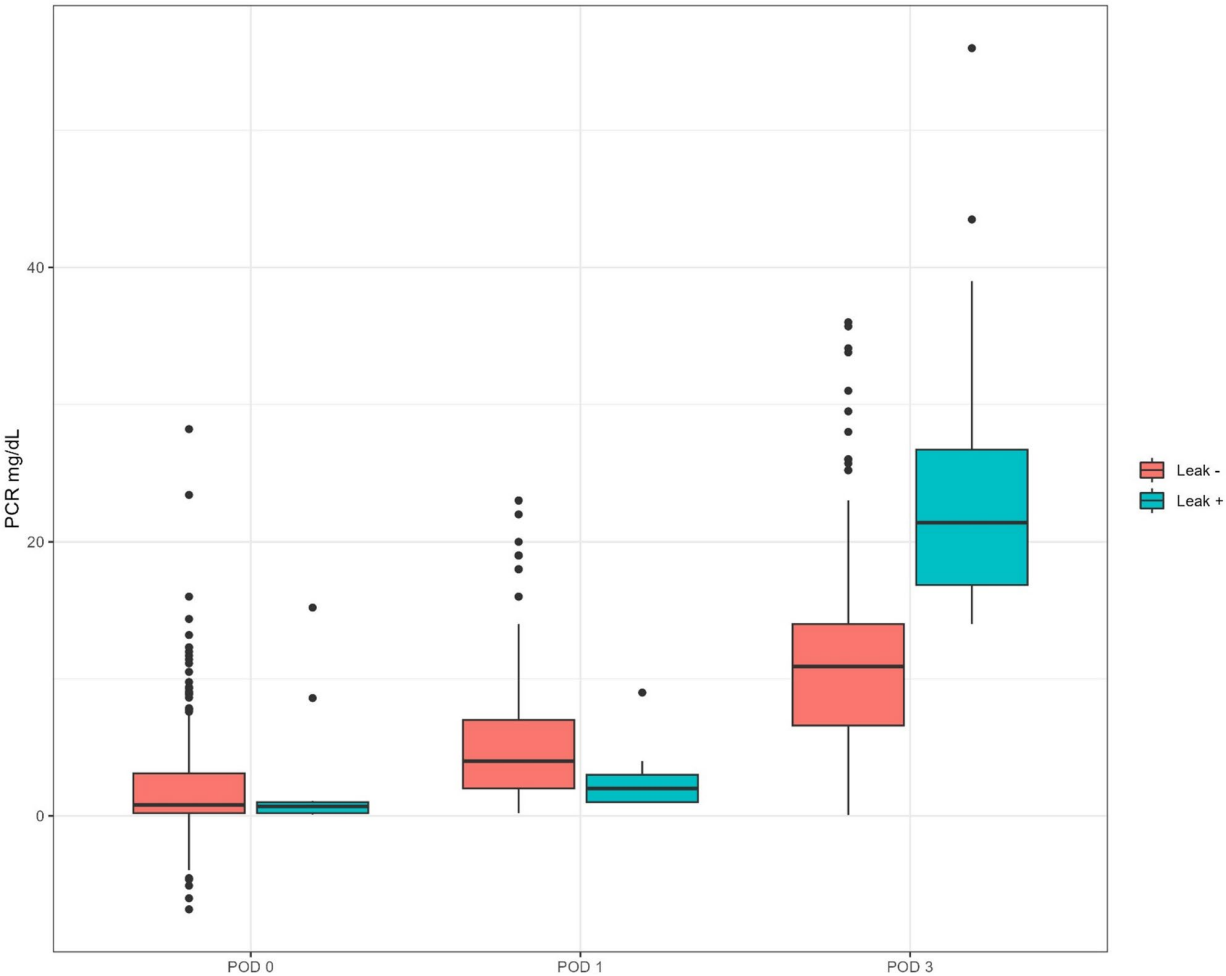
Boxplots showing C-reactive protein (CRP) levels (mg/dL) in patients with (Leak +) and without (Leak -) anastomotic leak at three postoperative days: POD 0, POD 1, and POD 3. At the start (POD 0), CRP levels were low and similar between groups. On POD 1, patients without leaks had a moderate increase in CRP, while those who later had a leak kept relatively low values. By POD 3, however, a clear difference appeared: patients with leaks had much higher CRP levels compared to the non-leak group. The boxplots show the median, interquartile range (IQR), and outliers, highlighting the wider distribution and higher upper quartiles in the leak-positive group on POD 3

**Fig. 2:**
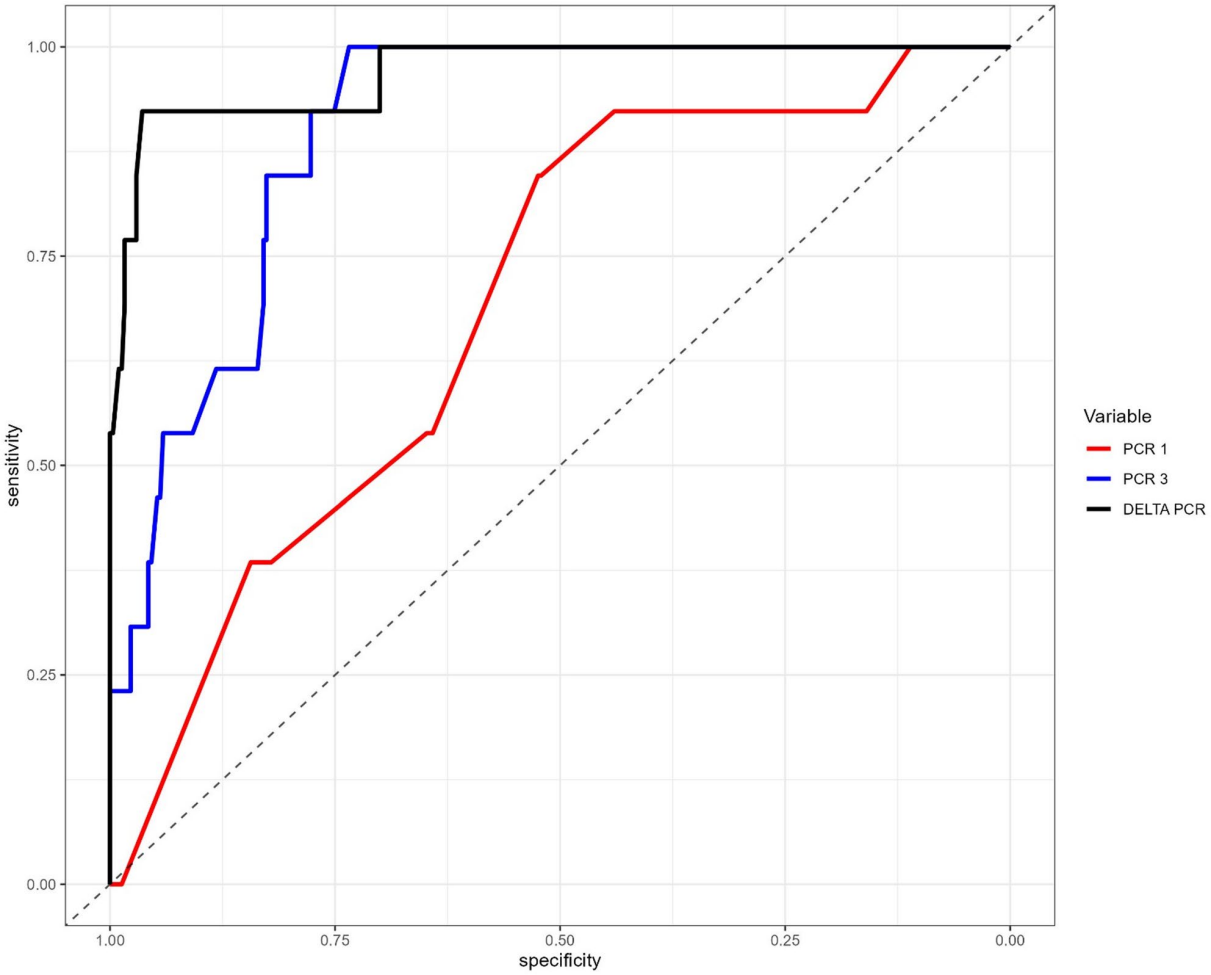
ROC curve analysis of CRP and ΔCRP for predicting anastomotic leak. Receiver operating characteristic (ROC) curves evaluate the diagnostic accuracy of CRP on POD 1 (red), CRP on POD 3 (blue), and the relative change in CRP (delta CRP, black) in identifying anastomotic leaks. The area under the curve (AUC) gives a measure of diagnostic performance, with higher values showing better differentiation ability. The figure indicates that CRP levels measured on POD 3, especially delta CRP, prove to be more sensitive and specific than CRP on POD 1, suggesting that both late postoperative CRP and its change over time are reliable indicators for early leak detection. The diagonal dashed line represents the performance of a random classifier (AUC = 0.5) and serves as a reference baseline

Postoperative CRP levels initially appeared higher in the NL group compared to the L group; conversely, at POD3 and for delta CRP, the levels were higher in the L group: these results are illustrated in the attached boxplots (Fig. 1). The predictive capacity of CRP values for the occurrence of a leak event was assessed using receiver operating characteristic (ROC) curves (Fig. 2). The POD1, POD3, and delta CRP variables demonstrated area under the curve (AUC) values of 0.692, 0.902, and 0.969, respectively, indicating that delta CRP is the most predictive measure compared to individual CRP values (Table 2). For delta CRP, the optimal cutoff was identified at 12.5 mg/dL, with a specificity of 0.964 (95% CI: 0.937–0.982) and a sensitivity of 0.923 (95% CI: 0.640–0.998; Table 2). The positive predictive value (PPV) of 0.522 (95% CI: 0.306–0.732) was low due to the low incidence of the event, which resulted in 11 false positives in this study (Table 3). Specifically, 12 events were correctly classified, 296 non-events were identified as negative, and a single event was misclassified as negative. The overall test accuracy was 0.963 (95% CI: 0.935–0.980). Odds ratios with their 95% confidence intervals and β coefficients for baseline CRP, POD1 CRP, POD3 CRP, and delta CRP, including both univariate and multivariate analyses, are explained in Supplementary Material 1: Table S4.

**Table 2:** Under the curve (AUC), sensitivity, specificity, accuracy and predictive positive value (PPV) of POD1, POD3 and CRP values

	AUC (95% C.I)	Trehshold	Specificity	Sensibility	Accuracy	PPV	NPV
CRP1	0.692 (0-561-0.823)	3.05	0.524 (0.467, 0.581)	0.846 (0.546, 0.981)	0.537 (0.481, 0.593)	0.070 (0.035, 0.122)	0.988 (0.956, 0.999)
CRP3	0.902 (0.848–0.956)	13.9	0.734 (0.681, 0.783)	1.000 (0.753, 1.000)	0.745 (0.694, 0.792)	0.138 (0.076, 0.225)	1.000 (0.984, 1.000)
Delta CRP	0.969 (0.9243-1)	12.5	0.964 (0.937–0.982)	0.923 (0.640–0.998)	0.963 (0.935–0.980)	0.522 (0.306–0.732)	0.997 (0.981 -1.000)

**Table 3:** Observed events in cohort, related to delta CRP

	Leak +	Leak -	Total
**Delta CRP Test +**	12	11	**23**
**Delta CRP Test -**	1	296	**297**
**Total**	**13**	**307**	**320**

## Discussion

AL represents an undesirable complication of all surgery, with effects on hospitalization, treatment costs, oncological prognosis, including effects on survival [1, 2]. Research of good markers is essential, because vital signs and majority of laboratory tests are slow in responding and are not sensitive for AL detection [4]. Delayed diagnosis is associated with an increase of mortality by 18% [5, 11]. In this direction, many authors demonstrated that CRP levels are more sensitive to detect surgical complications than clinical signs (pain, body temperature, heart rate), erythrocyte sedimentation rate (VES), and white blood cells (WBC) [11–13]. We stressed our evaluations on trend of CRP, in order to increase sensitivity and predictivity of the biomarker. This is one of the first prospective study on delta CRP, and it will be confirmed by more dedicated coohorts of colorectal patients. Nevertheless, regarding to CRP levels, because of the individual regulation of inflammatory responses, variability is frequent. Differences in postoperative day of analysis (from POD 1 to 7), in cut-off values (ranging from 94 to 190 mg/L), and in observed predictivity, are observed in literature [12]. Drugs, such as corticosteroids and statins, may also alter this response, which could alter the interpretation of data. The surgical approach may affect serum CRP levels [12]. Open surgery is associated with higher CRP profile than laparoscopic approach [17]. For the majority of authors, CRP on POD 3 had a good predictive value for infectious complications, especially AL [18]. After colorectal surgery, optimal cutoff value for CRP from 135 to 170 mg/L on POD 3 was found, while lower value (100 mg/L) was reported on POD 4 [19]. Endpoints of study and difference of cohorts are also determinant for statistical definition of cutoff [18].

A lot of metanalyses analyzed diagnostic accuracy of CRP [19]: it is significantly high for detecting after colorectal surgery in large series [13]. Moreover, these metanalyses displayed substantial heterogeneity (only 46% did not report heterogeneity among studies), indicating that these associations should be interpreted with caution [13, 14]. Regarding to time of CRP evaluation, different results are present in literature. In a recent prospective observational study, Baeza-Murcia et al. confirmed that CRP levels on POD 3 and 5 was useful for early detection also of asymptomatic AL [19]. However, Sala Hernandez et al. stressed the diagnostic value of POD 4 for excluding AL [20].

A reliable marker should be useful for early diagnosis of a severe complication like AL. CRP is synthesized by the liver within 6 h after stress events, reaching peak after about 48 h. Its plasmatic concentration is related to the acute stimulus [10, 11]. Advantage of acute phase proteins is that they increase before the onset of clinical signs, and this is more evident for those with early secretion, like CRP and procalcitonin [10]. Cost, repeatability and early analysis are good elements for a sensitive and specific marker. Recently, CRP was evaluated as a very specific marker that may exclude the event in the case of a negative test [13]. It maintains a potential role for indication to rule out early postoperative complications and AL. In this direction, with a good value of CRP a helpful for safe and early hospital discharge of the patient can be proposed [19].

Routine assessment of CRP levels as an additional complementary tool after colorectal surgery may be useful for predicting infectious complications and AL, reducing costs, as well as improving outcomes and patient care [13]. AL can be related to technical, vascular or mechanical etiology. Considering timing of diagnosis, we considered an early AL (POD1 to 4) and a late AL (from POD5) [2, 3, 6]. On this last type, clinical is less evident and radiologic findings are generally high sensitivity [6]. In POD 1, CRP is less sensitive because it reflects inflammatory response and pathogenesis of AL starts generally at 36–48 h from anastomosis [9, 11, 14]. According to CRP levels, we try to detect the most frequent type of AL, that occurred on POD3-4, identifying in early phase modification of inflammatory physiologic response [9]. Moreover, analysis In POD 1 and 3 with study of trend may be useful to improve sensitivity of this marker for early detection of AL, as our statistical analysis indicated. Our findings confirm recommendations for the diagnostic value of CRP for AL, with specific evaluation of the trend of this parameter as a prognostic tool. This is the first study with statistical analysis of delta CRP in oncological patients treated with colorectal resection. Novelty of results need of confirmation by other studies. Although scientific data are consistent and numerous evidence of value of delta CRP are weak, and our results should be necessarily evaluated with caution. If clinical evidence of this pilot study will be confirmed by more consistent statistical data, we should include delta CRP as a prognostic factor. Moreover, usefulness of delta CRP is strictly related to clinical prognosis, because may lead an alert on worsening of postoperative course. In fact, clinical course may differ from biochemical evolution: WBC, nutritional status and clinical signs revealed low levels of sensitivity [14]. We chose to study the first and third postoperative days because most fistulas occur starting from the third day postoperatively. A CRP assessment on the fifth day would not provide a real advantage in terms of treatment, as by that time, clinical assessment of the complication is usually already evident, and in many cases, diagnostic confirmation tests may be delayed. Some authors refer to preoperative CRP, particularly in evaluating the CRP/albumin ratio (CAR) [21]: however, the comparison between pre- and postoperative CRP is influenced by other factors, such as underlying inflammatory conditions, which could affect the prognostic value of the delta. Our aim, however, was to identify a clinical time point of inflammatory response variation that differs from the postoperative response and could help in further investigating AL. Costs, availability and comparability are resources of this biomarker. Similar results were observed for a cohort of patients with Crohn disease, for early detection of septic complications [15]. In this way, considering cancer an inflammatory chronic disease, CRP was confirmed in many trials as a prognostic tool [13, 19]. If we consider a lot of chronic preexisting disease and possible prediction of clinical course, delta CRP may act either excluding bias from abnormal basal value, either determining real worsening of clinical course, either solving limits of interindividual variability that determined various considered cut-off in literature [22]. In addition, all effects of type of surgery are reduced by analysis of delta CRP: lower values of laparoscopic patients do not affect cutoff with delta analysis. The different cutoffs validated by statistical studies from various research reflect the heterogeneity of the patient populations considered: for example, minimally invasive approaches tend to lead to a lower increase in CRP levels postoperatively. This did not result incident in our statistical evaluation. With delta PCR cut-off evidenced by our statistical analysis, we found high sensitivity and specificity of the markers, while PPV was limited by low events of AL observed in our cohort. Cohort size, number of events observed and statistical power are all factors that can consider our analysis a pilot study, but with very interesting prospective. Furthermore, evaluations at different postoperative days lead to variations in the cutoffs identified in the statistical analysis. These are also factors that support the prognostic validity of CRP variation, rather than the study of absolute values. We also performed the multivariate models, adjusted for BMI, age, and sex: they should be regarded only as sensitivity analyses to support the robustness of the findings, but they cannot be considered valid for rigorous inference.

Delta CRP may also be related to severity of AL, that Clavien-Dindo classification generally identifies with good sensitivity. In our study, no difference was observed for IIIb and Iva cases in terms of delta CRP, but we need of higher number of events to confirm a relation of delta CRP with severity. Moreover, analysis on gradation on severity was not primary endpoint.

Limits of our analysis are size of cohort, that can be extended to reinforce statistical results, and lack of evaluation of intraoperative complications; PPV should be recognized in larger cohort, to increase events of AL, to reinforce statistical value. In addition, analysis of subgroups of patients (laparoscopic, elderly) may add prognostic value for delta CRP, efforting statistical evidence on a specific onset. We must consider our clinical observation a good hypothesis for starting controlled randomized clinical trial on large scale, after good results of pilot studies like this.

In fact, increasing sample size is mandatory to confirm value of this biomarker, and we continue with enrolment. This may lead to a composite model of more biomarkers that will reinforce predictivity of delta CRP. Larger cohort, including more prognostic factor, controlled arm and randomization will be the keys to reinforce evidence of these preliminary data. Clinical impact of delta CRP, if confirmed by larger analysis, should be dramatic, in preclinical phase for an early approach to AL: surgical exploration should be more effective with limited abdominal compromission and fluid collection.

## Conclusions

Routine assessment of CRP levels as an additional complementary tool after colorectal surgery may be useful for predicting infectious complications and AL, reducing costs, as well as improving outcomes and patient care. Moreover, analysis in POD 1 and 3 with study of trend may be useful to improve sensitivity of this marker for early detection of AL, as our statistical analysis indicated. Our data confirmed that high NPV of postoperative CRP trend was able, already on POD 3, to rule out anastomotic complications, but value of these data is limited by low number of AL in our cohort. Delta CRP seems to increase sensitivity and specificity of this biochemical marker, which should be confirmed by larger cohorts. Although possible statistical value of delta CRP, limits of our analysis need of confirmation to impact clinical approach. Results are overall limited by the small sample size and low event rate. Moreover, we confirm exploratory nature of the findings, waiting for validation in larger cohorts. Cohort homogeneity and a comparison of the same endpoints would always be desirable for a correct interpretation of the data.

## Supplementary Information

Supplementary Material 1.

## Data Availability

The dataset analyzed in this study is not publicly available but is available to the corresponding author on reasonable request.

## Abbreviations

AL: Anastomotic leak
CRP: C-reactive protein
POD1: Postoperative day 1
L: leakage
NL: Non-leakage
AUC: Area under the curve
NPV: Negative predictive value
CT: Computed tomography
BMI: Body mass index
ASA score: American Society of Anesthesiologists
CCI: Charlson comorbidity index
